# Gut microbiota dysbiosis in sepsis and sepsis-associated organ injury: mechanisms, gut-organ axes, and therapeutic strategies

**DOI:** 10.3389/fmicb.2026.1886655

**Published:** 2026-07-21

**Authors:** Tingxi Zhang, Qian Wang, Wenli Qiu, Dingyu Zhang, You Shang

**Affiliations:** 1 Department of Critical Care Medicine, Union Hospital, Tongji Medical College, Huazhong University of Science and Technology, Wuhan, Hubei, China; 2 Department of Emergency and Critical Care Medicine, Tongji Hospital, Tongji Medical College, Huazhong University of Science and Technology, Wuhan, Hubei, China

**Keywords:** gut microbiota dysbiosis, host–microbe interactions, microbial metabolites, microbiota-targeted strategies, sepsis, sepsis-associated organ injury

## Abstract

Sepsis is a life-threatening syndrome caused by a dysregulated host response to infection and remains a major cause of morbidity, mortality, and organ dysfunction worldwide. Gut microbial dysbiosis in sepsis may result from both disease pathophysiology and ICU interventions, including broad-spectrum antibiotics, vasopressors, enteral or parenteral nutrition, mechanical ventilation, renal replacement therapy, immune status, and baseline comorbidities. Increasing evidence suggests that gut microbial dysbiosis is closely associated with sepsis progression and sepsis-associated organ injury, and may act as both a consequence of critical illness and a potential contributor to disease progression in selected experimental and clinical contexts. Host–microbe interactions, microbial metabolites, and immune-metabolic signaling help explain how gut dysbiosis contributes to sepsis pathophysiology. At the same time, probiotics, fecal microbiota transplantation, and selected microbial metabolites have shown possible benefits, mainly in experimental or selected clinical settings. This review integrates gut dysbiosis, microbial product translocation, microbial metabolites, and host–microbe interactions into a gut-organ axis framework, and evaluates how these mechanisms may contribute to sepsis progression, organ injury, and microbiota-targeted therapeutic strategies.

## 1 Introduction

Sepsis is a life-threatening syndrome caused by a dysregulated host response to infection and remains a leading cause of organ dysfunction and death worldwide (Evans et al., 2021). Although sepsis imposes a major clinical burden, treatment remains dominated by timely infection control, antimicrobial therapy, and organ support, with few interventions that directly modify the underlying host response (Adelman et al., 2020). For this reason, mechanisms that shape the host response have become important therapeutic targets beyond pathogen control and organ support.

The gut microbiota may contribute to the marked heterogeneity of sepsis progression and organ injury. Sepsis is frequently accompanied by profound gut microbiota dysbiosis, which may further aggravate disease progression, while sepsis itself exacerbates disruption of intestinal microbial homeostasis (Niu and Chen, 2021). Preclinical and clinical studies suggest that gut microbiota dysregulation is involved in sepsis-associated organ injury, including hepatic, renal, and other extraintestinal complications. These findings link the gut microbiota to both sepsis mechanisms and potential therapeutic strategies. This review integrates gut dysbiosis, microbial product translocation, microbial metabolites, and host–microbe interactions into a gut-organ axis framework to explain how intestinal microbial disruption may contribute to sepsis progression and organ injury. However, the causal direction of this relationship remains difficult to establish in human sepsis because microbiota alterations are strongly influenced by antibiotics, nutrition, immune status, and organ support (Baek et al., 2023). Therefore, this review distinguishes between causal-supporting experimental evidence, associative human evidence, and biologically plausible but still speculative mechanisms.

## 2 Literature search and evidence selection strategy

To improve transparency in literature selection, we conducted a structured literature search to identify studies relevant to gut microbiota dysbiosis, sepsis pathogenesis, sepsis-associated organ injury, and microbiota-targeted therapeutic strategies. PubMed and Web of Science were searched for English-language publications. The main search covered studies published up to December 2025.

Search terms included combinations of “sepsis,” “septic shock,” “critical illness,” “gut microbiota,” “gut microbiome,” “dysbiosis,” “intestinal barrier,” “bacterial translocation,” “microbial products,” “short-chain fatty acids,” “bile acids,” “fecal microbiota transplantation,” “probiotics,” “prebiotics,” “synbiotics,” “postbiotics,” “microbial metabolites,” “mycobiome,” “gut-lung axis,” “gut-kidney axis,” “gut-liver axis,” “gut-heart axis” and “gut-brain axis.” Additional topic-specific searches were performed for selected mechanisms and interventions discussed in the manuscript, including colonization resistance, epithelial tight junctions, regulated cell death, herbal and natural compounds.

Studies were first screened by title and abstract, followed by full-text assessment when they were relevant to the scope of this review. We included clinical studies, randomized controlled trials, systematic reviews, meta-analyses, animal studies, and mechanistic *in vitro* or ex vivo studies that addressed gut microbiota-related mechanisms in sepsis, critical illness, or sepsis-associated organ injury. Studies were excluded if they were not directly relevant to sepsis or critical illness, lacked sufficient mechanistic or clinical relevance, were not peer reviewed, or were available only as conference abstracts, editorials, or commentaries without original or synthesizable evidence.

The evidence base included clinical studies, animal models, mechanistic experiments, and emerging therapeutic platforms, we applied a tiered framework to interpret the literature according to both clinical relevance and level of causal support. Clinical studies, randomized trials, systematic reviews, and meta-analyses in sepsis or critically ill patients were given priority when available, but observational human studies were interpreted primarily as associative evidence rather than proof of causality, because gut microbiota composition in sepsis is heavily influenced by antibiotics, nutrition, ICU interventions, and disease severity, which can confound causal interpretation. Interventional animal studies, microbiota-transfer experiments, and experimental studies using defined microbial metabolites or pathway modulation were considered causal-supporting mechanistic evidence. *In vitro* studies, non-sepsis models, and indirect pathway-based findings were used mainly to support biological plausibility and were not presented as definitive evidence of clinical causality. Evidence concerning natural compounds, herbal formulations, and plant-derived nanovesicles was interpreted cautiously and considered primarily as preclinical or hypothesis-generating support. This framework was used to distinguish clinically supported findings from experimental mechanisms and speculative interpretations throughout the review. For representative studies that directly supported major mechanistic or therapeutic claims, we further extracted study-level information, including study design, model or population, microbiota-related exposure or intervention, major outcomes, principal findings, key limitations, and level of causal support. These details were incorporated into the relevant sections to avoid treating heterogeneous evidence as equivalent.

## 3 Gut microbiota dysbiosis in sepsis: ecological and metabolic features

**Figure 1:**
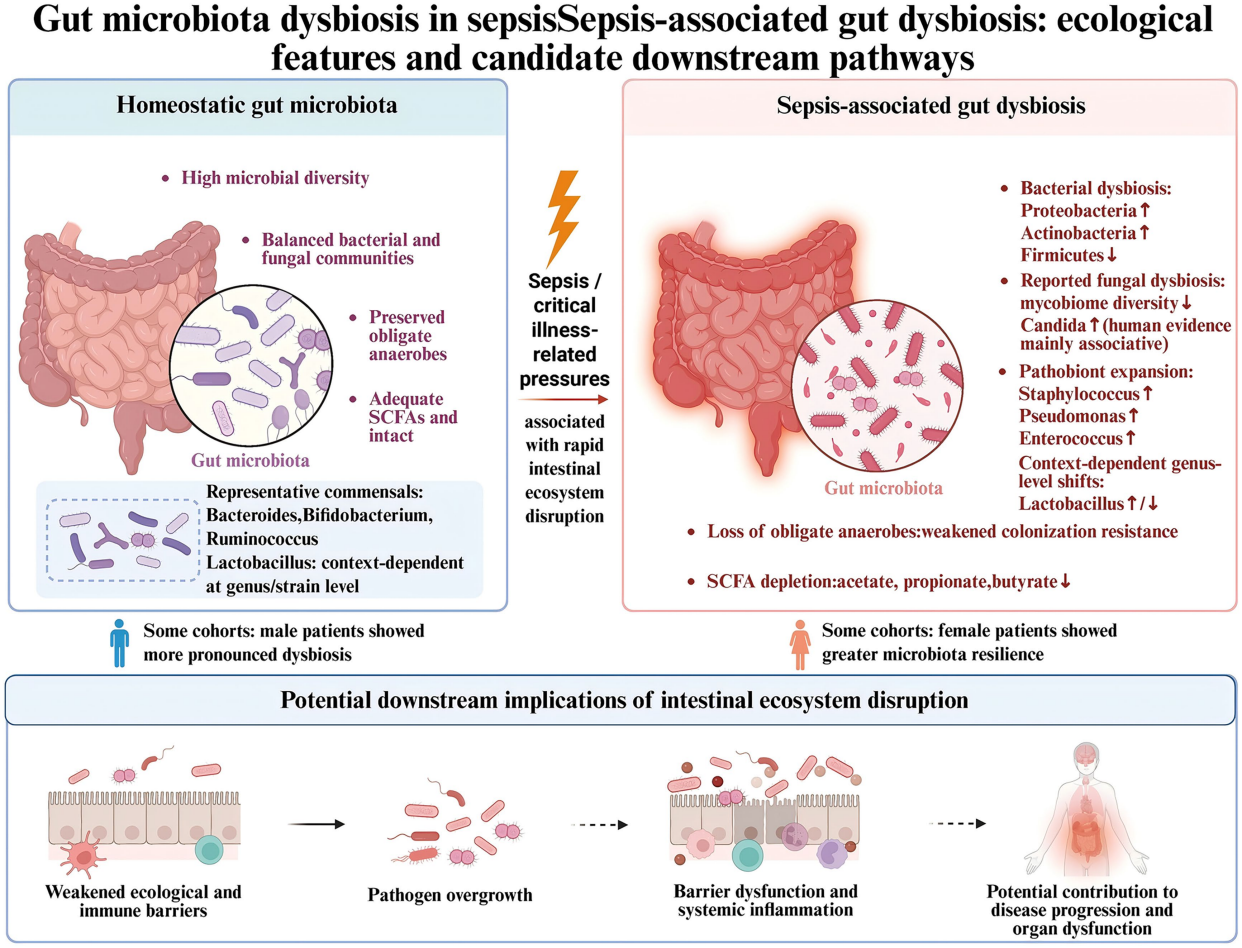
Gut microbiota dysbiosis in sepsis. Sepsis-associated gut dysbiosis: ecological features and candidate downstream pathways. Sepsis and critical illness-related pressures can rapidly disrupt the intestinal ecosystem, leading to reduced microbial diversity, depletion of obligate anaerobes and SCFA-producing taxa, pathobiont expansion, fungal imbalance, weakened colonization resistance, and reduced microbial metabolic output. These ecological and metabolic changes may weaken epithelial and immune barriers, promote pathogen overgrowth, amplify barrier dysfunction and systemic inflammation, and potentially contribute to disease progression and organ dysfunction. Solid arrows indicate pathways with stronger experimental or mechanistic support, whereas dashed arrows indicate associative, preclinical, or less validated candidate pathways.

Sepsis is accompanied by rapid changes in the gut microbiota, including loss of microbial diversity, depletion of obligate anaerobes and short-chain fatty acid (SCFA)-producing taxa, expansion of pathobionts, fungal imbalance, impaired colonization resistance, and reduced microbial metabolic output. This section outlines the physiological functions of the gut microbiota and the main features of sepsis-associated dysbiosis, as summarized in Figure 1.

### 3.1 Overview of the composition and function of the gut microbiota

Microbial communities vary along the gastrointestinal tract and are shaped by local physicochemical conditions. These include pH, oxygen tension, nutrients, and host-derived barriers (Yang et al., 2025a). Microbial diversity changes from proximal to distal intestine, with the distal colon having the highest cell density of mostly Firmicutes and Bacteroidetes comprising a high proportion of human microbiota. These bacteria contribute substantially to anaerobic fermentation and microbial metabolic output (Ruan et al., 2020; Yang et al., 2025a). In health, the gut microbiota supports host homeostasis by maintaining colonization resistance, producing bioactive metabolites, preserving epithelial barrier integrity, and shaping mucosal and systemic immune responses (Khan et al., 2021; Wu et al., 2021a; Liu et al., 2022).

These functions are particularly relevant to sepsis pathogenesis. Commensal anaerobes and SCFA-producing taxa help restrict pathobiont expansion, reinforce epithelial barrier function, and modulate immune responses (Ghosh et al., 2021; Khan et al., 2021; Caballero-Flores et al., 2023). Conversely, disruption of microbial homeostasis may weaken colonization resistance, impair barrier integrity, alter microbial metabolite availability, and amplify systemic inflammation (Adelman et al., 2020; Nedeva, 2021; Niu and Chen, 2021). In sepsis, the gut microbiota is more than a microbial ecosystem. It also acts as a barrier-related and immune-metabolic regulator that can influence infection progression and organ injury.

### 3.2 Characteristics of sepsis-associated gut microbiota dysbiosis

Gut microbial communities can change sharply during critical illness, and sepsis-associated dysbiosis may be detectable within hours of disease onset or ICU admission (Wozniak et al., 2022; Piccioni et al., 2024). Recent clinical microbiome studies provide study-level evidence for bacterial and fungal dysbiosis in sepsis. Park et al. (2024b) conducted a single-center prospective observational cohort study including 19 healthy controls, 18 sepsis patients, and 18 trauma patients, with stool samples collected on hospital days 14–21 for fungal internal transcribed spacer sequencing, they found a persistent shift from a diverse commensal mycobiome toward a *Candida*-dominated pathobiome: Basidiomycota was nearly depleted, Ascomycota became dominant, and Candida accounted for more than 95% of the average mycobiome abundance in sepsis patients, whereas *Penicillium* and *Saccharomyces* were nearly depleted, species-level analysis further suggested expansion of pathogenic Candida species, including *C. albicans* and *C. tropicalis*. Sun et al. (2023) performed a single-center observational study integrating 16S rRNA sequencing, fecal untargeted metabolomics, and CLP rat transcriptomics, after quality filtering, 38 sepsis patients and 19 healthy controls were included in the microbiome analysis, and 37 sepsis samples were included in metabolomic profiling, sepsis patients showed lower microbial diversity than controls (Shannon index 2.146 vs. 2.911, *p* < 0.01) and distinct microbial structure (ANOSIM *p* = 0.001), with enrichment of Proteobacteria, Enterococcaceae, *Lactobacillus*, *Enterococcus*, and *Klebsiella*, and depletion of Firmicutes, Lachnospiraceae, *Blautia*, Anaerostipes, *Bifidobacterium*, and *Eubacterium hallii* group. These studies support broad bacterial and fungal ecological disruption in sepsis, but their single-center observational design, limited sample size, antibiotic and ICU-related confounding, restricted sampling time points, and incomplete causal validation mean that these findings should be interpreted as associative rather than causal. Sepsis-associated microbial signatures should be interpreted with methodological heterogeneity in mind. Differences in 16S rRNA target regions, primers, sequencing platforms, OTU/ASV processing, reference databases, and bioinformatic pipelines may affect taxonomic assignment and diversity estimates, while shotgun metagenomics is influenced by sequencing depth, host DNA contamination, and reference genome coverage (Marascio et al., 2023). These factors may partly explain cross-study variability and limit direct reproducibility.

Compared with trauma patients, patients with sepsis exhibit more severe gut dysbiosis, with greater loss of microbial diversity and taxa. Sex-related differences have also been reported, with male patients showing more pronounced dysbiosis and female patients displaying greater resilience after trauma or sepsis (Munley et al., 2024; Park et al., 2024b). The clinical relevance of these shifts lies in their potential to erode colonization resistance and favor pathogen expansion. Severe systemic inflammatory response syndrome is associated with depletion of beneficial genera, including *Bifidobacterium* and *Lactobacillus*, and enrichment of potentially harmful taxa such as *Staphylococcus* and *Pseudomonas* (Shimizu et al., 2006). These divergent *Lactobacillus* findings likely reflect differences in cohort characteristics, disease stage, antibiotic exposure, ICU interventions, and taxonomic resolution. Because *Lactobacillus* is functionally heterogeneous, genus-level enrichment or depletion should be interpreted as species-, strain-, and context-dependent rather than uniformly beneficial or harmful (Al-Sadi et al., 2021; Fang et al., 2022). Moreover, mortality and infectious complications in critically ill patients have been associated with depletion of obligate anaerobes and expansion of potentially pathogenic bacteria (Shimizu et al., 2025). Loss of obligate anaerobes may therefore weaken colonization resistance and create ecological space for pathobiont expansion and infection (Khan et al., 2021; Caballero-Flores et al., 2023). This clinical evidence is important because it links ecological disruption with clinically relevant outcomes such as mortality and infectious complications, however, it remains observational and cannot determine whether obligate anaerobe depletion is a driver of poor outcome or a marker of disease severity, antibiotic exposure, and ICU-related ecological pressure.

Sepsis-associated dysbiosis is also accompanied by metabolic disruption. Intestinal SCFA levels are reduced in sepsis, and SCFA availability may be further compromised during critical illness (Valdés-Duque et al., 2020; Lou et al., 2022). Reduced SCFA availability in sepsis may aggravate epithelial barrier dysfunction, weaken colonization resistance, and impair recovery from critical illness, as SCFAs support epithelial energy metabolism, regulate epithelial proliferation and differentiation, and modulate local and systemic immunity (Deleu et al., 2021; Ney et al., 2023). These findings show that sepsis disrupts microbial diversity, anaerobic dominance, colonization resistance, and metabolic output at the same time. Whether these changes initiate sepsis progression or mainly reflect disease severity remains unresolved. Therefore, human microbiome data should be interpreted primarily as associative unless supported by longitudinal sampling, microbial transfer experiments, or intervention studies. Nevertheless, these changes may help identify patients with impaired colonization resistance and a higher risk of secondary infection.

## 4 Microbial and host mechanisms linking gut dysbiosis to sepsis pathogenesis

**Figure 2:**
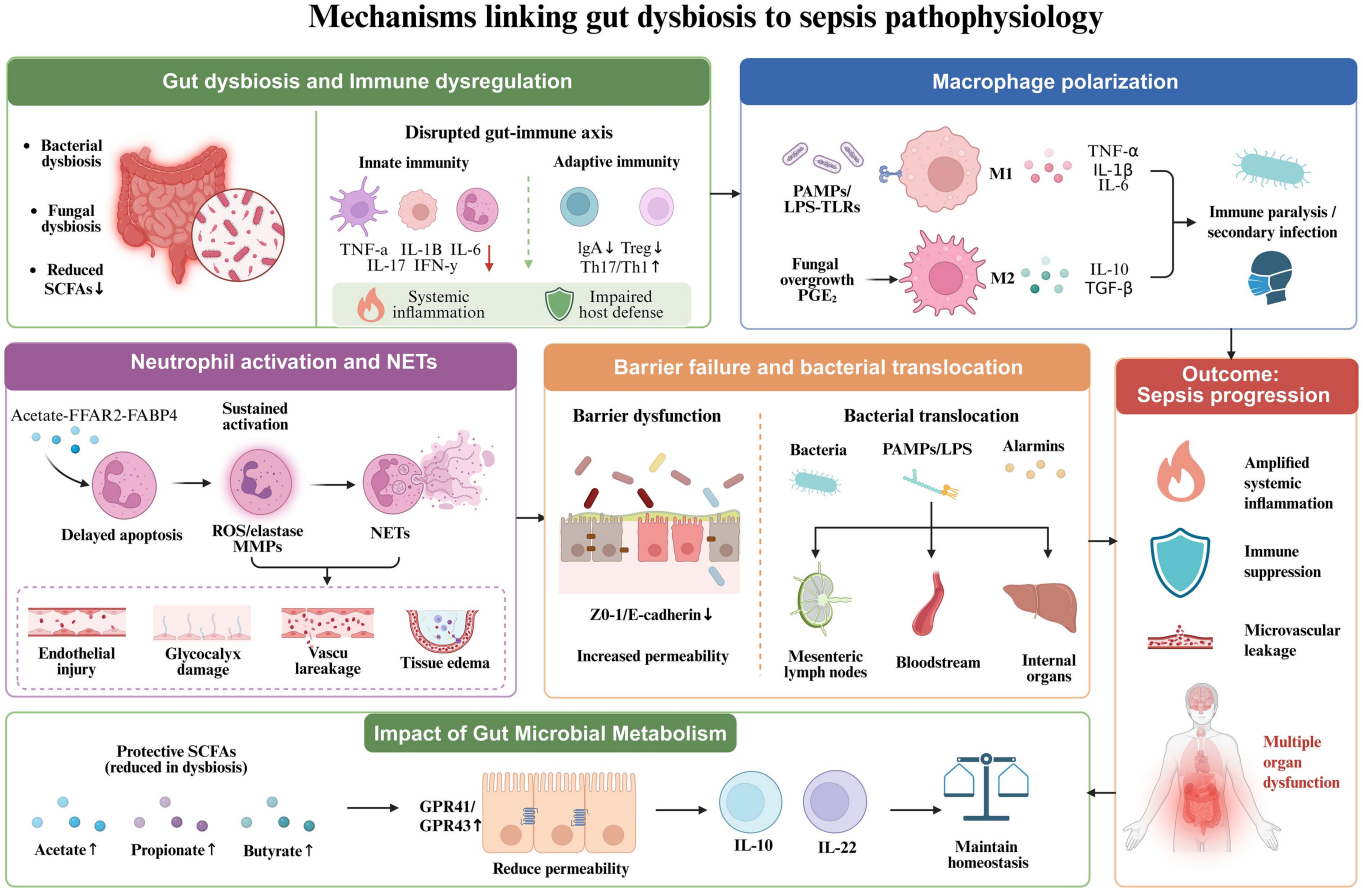
Microbial and host mechanisms linking gut dysbiosis to sepsis pathogenesis. Gut microbiota dysbiosis promotes sepsis progression through immune, barrier, and metabolic mechanisms. Disruption of the gut-immune axis alters macrophage polarization, enhances neutrophil activation, and promotes excessive neutrophil extracellular trap formation, thereby amplifying inflammatory and vascular injury. Epithelial barrier failure facilitates bacterial translocation and systemic exposure to pathogen-associated molecular patterns, bacteria, and other luminal components. Dysbiosis also alters microbial metabolite profiles, particularly SCFAs, affecting epithelial integrity, immune regulation, and systemic inflammatory balance.

Based mainly on experimental and mechanistic studies, gut dysbiosis may influence sepsis progression through several interconnected processes, including immune dysregulation, epithelial barrier failure, and microbial product exposure. These shared mechanisms provide the basis for the organ-specific gut-organ axis injuries discussed below and are summarized in Figure 2. In human sepsis, however, these pathways should be interpreted as mechanistically plausible rather than definitively causal.

### 4.1 Immune dysregulation and systemic inflammation

Under physiological conditions, gut microbes continuously educate mucosal and systemic immunity while preserving intestinal immune tolerance (Zheng et al., 2020; Wiertsema et al., 2021). In sepsis, microbial dysbiosis disrupts the gut-immune axis and contributes to dysregulated innate immune responses, systemic inflammation, and impaired host defense (Niu and Chen, 2021; Schlechte et al., 2023; Xu, 2025).

At the cellular level, gut dysbiosis may reshape septic immune responses through several interconnected immune cell programs, particularly macrophage polarization, neutrophil activation, NET formation, and metabolite-dependent immune regulation.

Macrophages are an important cellular node in disrupted gut-immune communication. In the initial stages of infection, these immune cells identify pathogen-associated molecular patterns (PAMPs), such as lipopolysaccharides (LPS) from Gram-negative bacteria, via Toll-like receptors (TLRs), which triggers the activation of innate immune responses (Kumar, 2020; Zamyatina and Heine, 2020). As sepsis advances, the phenotypes of macrophages may undergo dynamic remodeling, transitioning in certain contexts from pro-inflammatory programs to states associated with anti-inflammatory or tissue repair (Chen et al., 2021). Evidence from a non-sepsis experimental model suggests that antibiotic-induced dysbiosis can promote intestinal fungal overgrowth and fungi-induced PGE2 production, thereby favoring M2 macrophage polarization (Wu et al., 2021b; Al-Rashidi, 2022). This pathway provides a plausible mechanism by which dysbiosis may influence macrophage phenotypes, but its relevance to human sepsis remains to be established. In sepsis models, anti-inflammatory or M2-like macrophage polarization has been implicated in sepsis-induced immunosuppression, partly through mediators such as IL-10 and TGF-β (Wang and Wang, 2023). Experimental studies outside classical sepsis models suggest that SCFAs can regulate macrophage antibacterial responses through LAMTOR2-dependent signaling and enhance activated CD8^+^ T-cell function through metabolic and epigenetic reprogramming (Wu et al., 2020; Luu et al., 2021).

Neutrophils provide another route through which gut dysbiosis may translate into systemic inflammatory injury. Neutrophils are indispensable for pathogen control, but persistent or poorly regulated activation can convert them into drivers of inflammatory tissue injury during sepsis (Zhang et al., 2025). In a study combining sepsis-induced ARDS patient samples and CLP-induced septic mice, gut microbiota-derived acetate was shown to delay neutrophil apoptosis through an FFAR2-FABP4-endoplasmic reticulum stress pathway (Xuan et al., 2024). Although direct evidence in sepsis remains limited, studies in severe inflammatory and critical illness settings suggest that fungal dysbiosis, particularly *Candida* expansion, may sustain neutrophil activation (Kusakabe et al., 2023). Excessively activated neutrophils release reactive oxygen species (ROS) and proteolytic enzymes, including elastase and matrix metalloproteinases (MMPs), thereby promoting endothelial injury, extracellular matrix degradation, microvascular leakage, and progression toward systemic inflammation and multiple organ dysfunction (Shen et al., 2021; Liu et al., 2023; Zhang et al., 2025). In the context of gut dysbiosis, these pathways should be interpreted as downstream inflammatory mechanisms that may be modulated by microbial or metabolic signals, rather than as direct evidence that dysbiosis itself causes neutrophil-mediated organ injury. Thus, gut dysbiosis may influence neutrophil-driven injury not as an isolated trigger, but by altering microbial and metabolic signals that regulate neutrophil survival, activation, and endothelial damage.

Neutrophil extracellular traps (NETs) further link antimicrobial defense to vascular injury. Although NETs help trap and neutralize pathogens, excessive NET formation can damage the endothelial glycocalyx, disrupt intercellular junctions, upregulate adhesion molecule expression, and promote endothelial apoptosis, thereby increasing vascular permeability, promoting tissue edema, aggravating hypotension, and contributing to organ dysfunction in sepsis (Maneta et al., 2023; Zhang et al., 2023a; Pignataro et al., 2025).

Beyond its effects on innate immune responses, gut dysbiosis also alters microbiota-derived metabolic signals. In particular, dysbiosis reduces the production of beneficial metabolites such as SCFAs (Adelman et al., 2020; Zhou and Liao, 2021). Experimental and review-based evidence from intestinal barrier and infection-related models suggests that acetate, propionate, and butyrate can support epithelial barrier integrity by modulating tight junction proteins and GPR41/GPR43-related signaling (Pérez-Reytor et al., 2021; Caetano and Castelucci, 2022; Tang et al., 2025a). SCFAs also act as immune-regulatory signaling molecules. In cases of sepsis-associated encephalopathy induced by cecal ligation and puncture (CLP), the administration of SCFAs decreases mortality rates, enhances the integrity of both the intestinal and blood–brain barriers, and mitigates neuroinflammation (Liu et al., 2021b; Lou et al., 2022). Evidence from non-sepsis intestinal immune models suggests that SCFAs can modulate mucosal immunity by promoting IL-10-related anti-inflammatory responses and inducing IL-22 production from CD4^+^ T cells and innate lymphoid cells (Park et al., 2015; Wiertsema et al., 2021; Ney et al., 2023). These findings indicate that microbial immune and metabolic signals can shape the septic response, either supporting host defense or promoting inflammatory injury and immune dysfunction.

### 4.2 Intestinal barrier dysfunction and bacterial translocation

#### 4.2.1 Barrier architecture and homeostatic function

The intestine is both an absorptive organ and a highly regulated immune barrier (Wiertsema et al., 2021). One of its core functions is to restrict the entry of luminal microbes and microbial products into systemic compartments (Schoultz and Keita Å, 2020; Oami et al., 2025). Epithelial cells are linked by junctional complexes, including tight junctions, adherens junctions, and desmosomes (Schoultz and Keita Å, 2020; Raya-Sandino et al., 2021). Key tight junction proteins include claudins, occludin, junctional adhesion molecules, and cytosolic scaffolds such as ZO-1, ZO-2, and ZO-3 (Moonwiriyakit et al., 2023). In adherens junctions, afadin links nectin to the actin cytoskeleton, whereas catenin connects E-cadherin to actin (Lessey et al., 2022).

#### 4.2.2 Critical illness associated barrier failure

Critical illness can impair the intestinal barrier and promote gut-derived systemic inflammation and multiple organ dysfunction syndrome (MODS) (Oami et al., 2025). In a burn injury model, gut microbiota composition changed rapidly after systemic injury, with increased Gram-negative aerobic bacteria, Enterobacteriaceae expansion, increased intestinal permeability, and bacterial translocation to mesenteric lymph nodes within 1 day (Earley et al., 2015). These findings suggest that the gut can act as a reservoir for bacterial dissemination after severe injury, contributing to sepsis and potentially MODS (He et al., 2019). In a severe burn injury mouse model, intestinal SCFA levels were reduced in parallel with gut microbiota disruption and impaired barrier function (Feng et al., 2019).

Intestinal barrier disruption and bacterial translocation are increasingly recognized as important amplifiers of sepsis pathogenesis. When barrier integrity is compromised, luminal bacteria, PAMPs, alarmins, and other microbial products may gain access to systemic immune compartments, thereby amplifying inflammation and organ dysfunction (Di Vincenzo et al., 2024). Several host epithelial pathways further modulate the severity of sepsis-associated barrier leakage and determine whether intestinal injury progresses to systemic microbial product exposure. Gong et al. (2025) investigated the ACE2–microbiota–5-MTP axis using 71 human sepsis samples, CLP- and *E. coli*-induced sepsis models, ACE2 knockout or overexpression mice, FMT, microbiome–metabolome profiling, 5-MTP supplementation, and Caco-2 experiments, clinically, lipopolysaccharide-binding protein predicted in-hospital death with moderate accuracy (AUC = 0.734, *p* = 0.002) and correlated strongly with serum ACE2 levels (*r* = 0.737, *p* < 0.001), experimentally, ACE2 deficiency worsened intestinal permeability, bacterial translocation, organ injury, and 7-day mortality after CLP, reducing survival from 52% in WT-CLP mice to 20% in KO-CLP mice (*p* = 0.0012), whereas 5-MTP supplementation partly restored epithelial repair through PI3K-AKT-WEE1 signaling. However, the human data are mainly correlative and based on a limited sample size, without direct clinical validation of the ACE2-microbiota-5-MTP-gut barrier causal axis. Thus, although the mechanism is well supported by animal and cellular evidence, its clinical relevance still requires further confirmation. Hu et al. (2019) investigated the mtDNA–STING pathway using intestinal biopsies from five patients with abdominal sepsis and enterocutaneous fistula, together with CLP-induced sepsis models, STING knockout mice, STING agonist treatment, mtDNA stimulation, and DNase I intervention, in human samples, intestinal STING expression was increased and correlated with epithelial apoptosis (*R*^2^ = 0.74, *p* = 0.01) and serum I-FABP levels (*R*^2^ = 0.511, *p* = 0.02), in CLP mice, STING knockout reduced intestinal inflammation, permeability, bacterial translocation, and organ injury, improving 120-h survival from 25% in WT-CLP mice to 55% in STING-KO mice, whereas STING activation with 5,6-dimethylxanthenone-4-acetic acid (DMXAA) reduced survival to 0% (*p* = 0.0345). Although the study provides a coherent mechanistic link between mtDNA-STING signaling and sepsis-induced gut barrier injury, its clinical evidence is limited by the very small, disease-specific human cohort and mainly correlative design, requiring further validation for broader clinical translation.

Qian et al. (2025) used CLP-induced abdominal sepsis mice, FXR agonist INT-747, intestinal epithelial cell-specific *Myd88* deficiency, FMT, 16S rRNA sequencing, bile acid profiling, and probiotic intervention to examine the dysbiosis–Myd88–FXR/FGF15 axis; CLP induced gut barrier injury, cholestasis, FXR/FGF15 suppression, and microbiota disruption, with 24-h and 48-h survival rates of 65 and 50%, respectively; Functionally, intestinal epithelial MyD88 deficiency preserved FXR/FGF15 signaling, improved bile acid homeostasis, reduced bacterial translocation, and increased 30-day survival from 10 to 40%. Because cholestasis is linked to poor outcomes in sepsis, this pathway may be relevant to disease severity (Juschten et al., 2022; Tanaka et al., 2022; Xu et al., 2025). By linking dysbiosis to MyD88-FXR/FGF15-mediated barrier dysfunction, this work offers a plausible mechanistic model; however, its dependence on murine models, short-term interventions, and absence of human validation restricts direct clinical translation. Fredenburgh et al. (2011) used COX-2-deficient and wild-type CLP mice, bone marrow chimeric models, and C2BBe1 intestinal epithelial cells to examine the role of epithelial COX-2 in sepsis-induced gut barrier dysfunction, after CLP, ileal COX-2 expression increased approximately 3-fold at the mRNA level and more than 20-fold at the protein level in wild-type mice, COX-2 deficiency worsened sepsis outcomes, with 80% mortality by day 4 compared with 30% mortality by day 5 in wild-type mice, more severe hypotension at 72 h after CLP (57 ± 4 vs. 73 ± 2 mmHg), increased bacterial translocation, and greater intestinal permeability (>4-fold vs. 1.2-fold increase in serum FD-4); mechanistically, COX-2 deficiency or inhibition reduced ZO-1, occludin, and claudin-1 expression, whereas COX-2-derived PGD₂ attenuated cytokine-induced epithelial hyperpermeability *in vitro*. Although these findings support a protective COX-2-PGD2 axis in sepsis-induced gut barrier dysfunction, the lack of human validation and reliance on global knockout and pharmacological inhibition models limit direct clinical translation. Evidence from a non-sepsis intestinal barrier-defect model suggests that increased intestinal permeability can promote systemic inflammation, disturb iron recycling, and create an iron-rich environment that favors bacterial growth and increases susceptibility to sepsis (Cherayil, 2011; Kumar et al., 2020). This provides mechanistic support for a barrier failure-iron metabolism-infection axis, but it remains extrapolated experimental evidence rather than direct human sepsis evidence. Overall, barrier disruption links gut dysbiosis to sepsis mainly by increasing systemic exposure to microbial products, while ACE2/5-MTP, STING, FXR-FGF15, and COX-2-related pathways may modulate the severity of epithelial leakage and inflammatory amplification.

### 4.3 Microbial products, pathobionts, and host barrier disruption

Loss of colonization resistance provides a microbial basis for barrier failure and systemic inflammatory amplification in sepsis. Under healthy conditions, commensal anaerobes limit pathogen expansion through nutrient competition, antimicrobial metabolite production, and immune conditioning (Khan et al., 2021; Caballero-Flores et al., 2023). During sepsis and critical illness, depletion of obligate anaerobes and SCFA-producing taxa can create ecological space for pathobiont expansion, weakening both microbial and epithelial barriers (Caballero-Flores et al., 2023; Ney et al., 2023; Shimizu et al., 2025). This shift increases the likelihood that microbial products or viable bacteria will cross the mucosal barrier and engage host inflammatory pathways.

Microbial products are key mediators linking dysbiosis to host injury. Experimental and translational studies support the concept that disruption of epithelial barrier integrity may allow PAMPs such as LPS and other luminal microbial products to gain increased access to systemic compartments, where they can activate pattern recognition receptors on innate immune cells (Kumar, 2020; Zamyatina and Heine, 2020; Di Vincenzo et al., 2024). This process amplifies cytokine production, endothelial injury, vascular leakage, and organ dysfunction. In this sense, gut dysbiosis contributes to sepsis pathogenesis not only by altering community composition, but also by increasing host exposure to microbial ligands capable of sustaining systemic inflammation.

In addition to classical microbial products such as LPS, specific microbiota-derived metabolites can directly damage epithelial junctions. Ma et al. (2022) investigated the CYP1A1–microbiota–cadaverine axis using MRSA-induced abdominal sepsis mice, CYP1A1 knockout, RNA-seq, 16S rDNA sequencing, targeted metabolomics, cadaverine supplementation, *Enterococcus faecalis* gavage, antibiotic-induced dysbiosis, and clinical samples from 18 septic patients with persistent MRSA infection and 20 healthy controls, *cyp1a1* deficiency improved survival after MRSA challenge compared with wild-type mice (HR = 4.739, 95% CI 1.23–18.25, *p* = 0.003), reduced blood bacterial load (*p* = 0.002), preserved ZO-1, occludin, and E-cadherin expression, and decreased intestinal permeability, mechanistically, Cyp1a1 deficiency reduced cadaverine production, whereas cadaverine supplementation or *E. faecalis* gavage reversed its barrier-protective effect; *E. faecalis* abundance strongly correlated with cadaverine levels (*r* = 0.9123, *p* < 0.0001), in patients, fecal cadaverine positively correlated with SOFA score (*r* = 0.8114, *p* < 0.0001). This study identifies a CYP1A1-microbiota-cadaverine–epithelial junction axis in MRSA-induced abdominal sepsis, but its clinical relevance remains limited by small correlative patient data and reliance on global knockout and microbiota-intervention models. These changes provide a mechanistic link between microbial metabolic dysregulation and junctional barrier failure.

Certain enteric pathobionts can also directly promote barrier disruption and bacterial dissemination. Bacterial translocation refers to the passage of intestinal bacteria across the mucosal barrier into normally sterile sites, including mesenteric lymph nodes and extraintestinal organs, and may represent a key step in the escalation of sepsis (Potruch et al., 2022; Charitos et al., 2025). Wallaeys et al. (2024) used a tumor necrosis factor (TNF)-induced lethal sepsis-like mouse model, Paneth cell-specific P55 deletion, IFNAR1 deficiency, microbiota depletion, Paneth cell RNA-seq/proteomics, and UPR reporter assays to show that TNF-P55 signaling activates type I IFN responses and suppresses IRE1-dependent UPR in Paneth cells, thereby reducing antimicrobial peptide production and promoting gut bacterial translocation, polymicrobial sepsis, organ injury, and death. Although this study defines a coherent TNF-P55-IFN-UPR-Paneth cell dysfunction axis, its reliance on acute TNF administration and lack of validation in classical infection-induced sepsis models or human samples limit direct clinical translation. Beyond host-mediated defects in mucosal defense, selected enteric pathogens can directly degrade epithelial junctions and thereby facilitate translocation (Paradis et al., 2021; Zheng et al., 2021). Among them, *Aeromonas sobria* can disseminate beyond the intestine in immunocompromised or older individuals and cause extraintestinal infections, including sepsis (Kobayashi et al., 2019; Das et al., 2022). Its secreted serine protease degrades multiple adherens and tight junction components, including nectin-2, afadin, ZO-1, ZO-2, ZO-3, and claudin-7, leading to barrier breakdown and systemic spread (Kobayashi et al., 2019; Ueda et al., 2022).

These findings support a feed-forward loop in sepsis: dysbiosis weakens colonization resistance and barrier integrity, microbial products and pathobionts worsen inflammatory and junctional injury, and barrier failure further increases host exposure to gut-derived microbial stimuli. Direct causal evidence is strongest for barrier disruption, microbial product exposure, and selected metabolite-mediated pathways, whereas fungal dysbiosis and some pathobiont-specific mechanisms remain less well validated in human sepsis.

## 5 Role of gut microbiota in sepsis-associated organ injury

**Figure 3:**
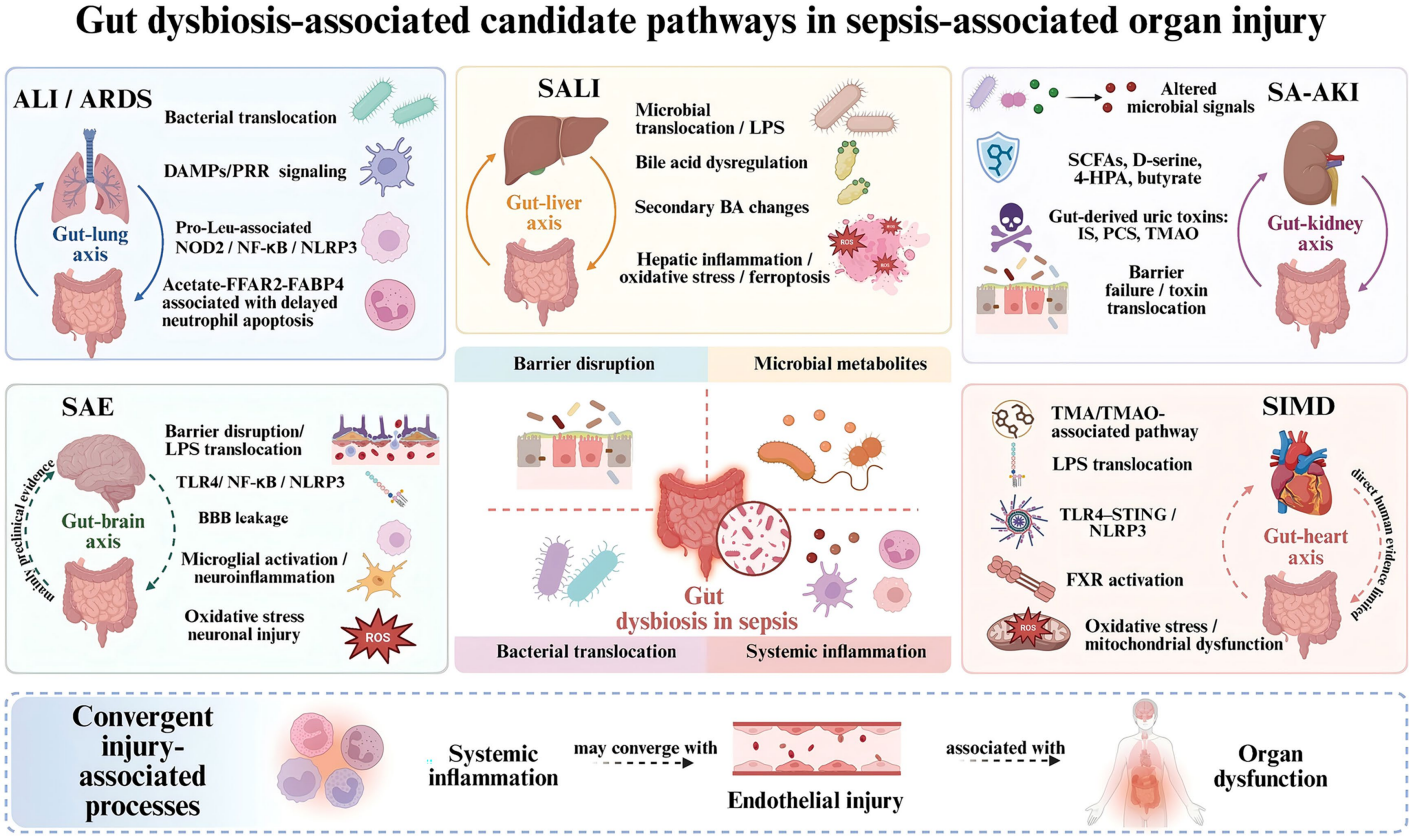
Gut dysbiosis-associated candidate pathways in sepsis-associated organ injury. Gut dysbiosis-associated candidate pathways in sepsis-associated organ injury. Gut microbiota dysbiosis may contribute to sepsis-associated ALI/ARDS, SALI, SA-AKI, SAE, and SIMD through organ-specific gut–organ axes. Shared mechanisms include epithelial barrier disruption, bacterial or microbial product translocation, altered microbial metabolites, systemic inflammation, and endothelial injury. These processes may involve pathway-specific signals such as Pro-Leu-NOD2/NF-κB/NLRP3 and acetate-FFAR2-FABP4 signaling in ALI/ARDS; LPS translocation, bile acid dysregulation, oxidative stress, and ferroptosis in SALI; SCFAs, D-serine, 4-HPA, butyrate, and gut-derived uremic toxins in SA-AKI; LPS–TLR4/NF-κB/NLRP3 signaling, BBB leakage, microglial activation, neuroinflammation, and neuronal injury in SAE; and TMA/TMAO-associated pathways, LPS translocation, FXR activation, oxidative stress, and mitochondrial dysfunction in SIMD. These pathways may converge on systemic inflammation and endothelial injury, ultimately promoting organ dysfunction. The mechanisms shown are candidate pathways with heterogeneous levels of supporting evidence. Solid arrows indicate pathways with stronger experimental or mechanistic support, whereas dashed arrows indicate associative, preclinical, or less validated candidate pathways.

Across these organ axes, gut dysbiosis appears to act through several shared routes, including barrier failure, microbial product translocation, altered microbial metabolites, and inflammatory signaling. Organ-specific injury then depends on how these shared signals interact with local immune, vascular, metabolic, and parenchymal responses. This section summarizes the mechanisms by which gut dysbiosis contributes to sepsis-associated lung, kidney, liver, cardiac injury and encephalopathy through organ-specific gut-organ axes. (Figure 3; Table 1). This gut-organ axis framework is also supported by recent reviews emphasizing that gut-derived microbial translocation, endotoxins, and altered metabolites may affect the lung, kidney, liver, brain, and heart through overlapping inflammatory and metabolic pathways during sepsis (Wang et al., 2025). Because the evidence supporting each gut-organ axis differs substantially, the organ-axis mechanisms discussed below should not be interpreted as equally mature. Some pathways are supported by human observational cohorts and experimental intervention studies, whereas others rely mainly on animal models, metabolomic associations, *in vitro* mechanistic experiments, or extrapolation from non-sepsis settings. To reflect this heterogeneity, Table 1 includes an evidence-grading column indicating the main type and strength of evidence supporting each pathway.

**Table 1:** Gut microbiota-mediated mechanisms in sepsis-associated organ injury.

Organ injury	Gut-organ axis	Microbiota-related alterations	Key mediators/pathways	Evidence grading	Pathophysiological consequences	References
ALI/ARDS	Gut-lung axis	Barrier disruption; bacterial translocation; altered microbial metabolites	DAMPs/PRRs; Pro-Leu-NOD2/NF-κB/NLRP3; acetate-FFAR2-FABP4	Animal sepsis/ALI models; *in vitro* neutrophil mechanisms; selected human translational data for sepsis-induced ARDS	Pulmonary inflammation; epithelial injury; increased vascular permeability	Zhou and Liao (2021); Xuan et al. (2024); Yang et al. (2025b)
SA-AKI	Gut-kidney axis	Dysbiosis; barrier failure; altered microbial metabolites	SCFAs; D-serine; IS/PCS/TMAO; STING-GSDMD; NOX2-dependent T-cell activation	Animal SA-AKI models; cell-based renal tubular mechanisms; metabolomics/uremic toxin associations; limited human AKI cohort evidence	Tubular injury; renal inflammation; pyroptosis; renal dysfunction	Nakade et al. (2018); Dicu-Andreescu et al. (2024); Tian et al. (2025)
SALI	Gut-liver axis	Portal translocation of gut-derived microbes/LPS; bile acid dysregulation	LPS-mediated inflammation; BAs dysregulation; oxidative stress	Animal SALI models; metabolomics and microbiota studies; hepatocyte mechanistic experiments; limited human observational evidence	Hepatic inflammation; hepatocellular injury; bile acid accumulation; endotoxemia	Sun et al. (2020); Larabi et al. (2023) Punzo et al. (2024)
SAE	Gut-brain axis	Dysbiosis with barrier failure, microbial metabolite depletion, and pathobiont translocation	LPS-TLR4-NF-κB-NLRP3; SCFA-GPR43/GPR109A-Nrf2; AhR signaling; BBB disruption	Animal SAE models; mechanistic preclinical evidence; limited human association	BBB leakage, neuroinflammation, oxidative stress, neuronal injury, cognitive dysfunction	Yoseph et al. (2016); Fang et al. (2022); Liao et al. (2022); Tan et al. (2022b); Li et al. (2025)
SIMD	Gut-heart axis	LPS translocation; altered TMA/TMAO and BA metabolism; selected taxa enrichment	TMAO; LPS-TLR4; STING/NLRP3; FXR; mitochondrial dysfunction	Human microbiota association studies; animal/in vitro myocardial injury mechanisms	Myocardial inflammation; apoptosis; conduction abnormalities; impaired contractility	Chen et al. (2022); Deng et al. (2023); Zhang et al., (2023b)

This table summarizes organ-specific mechanisms linking gut microbiota dysbiosis to sepsis-associated lung, kidney, liver, and cardiac injury. Key mechanisms include barrier disruption, microbial translocation, altered microbial metabolites, immune dysregulation, oxidative stress, and organ-specific inflammatory signaling. ALI, acute lung injury; ARDS, acute respiratory distress syndrome; SA-AKI, sepsis-associated acute kidney injury; SALI, sepsis-associated liver injury; SAE, sepsis-associated encephalopathy; SIMD, sepsis-induced myocardial dysfunction; DAMPs, damage-associated molecular patterns; PRRs, pattern recognition receptors; Pro-Leu, proline-leucine; NOD2, nucleotide-binding oligomerization domain-containing protein 2; NF-κB, nuclear factor-κB; NLRP3, NOD-like receptor family pyrin domain-containing 3; FFAR2, free fatty acid receptor 2; FABP4, fatty acid-binding protein 4; SCFAs, short-chain fatty acids; IS, indoxyl sulfate; PCS, p-cresyl sulfate; GPR, G protein-coupled receptor; BBB, blood–brain barrier; TMAO, trimethylamine N-oxide; STING, stimulator of interferon genes; GSDMD, gasdermin D; NOX2, NADPH oxidase 2; LPS, lipopolysaccharide; BAs, bile acids; FXR, farnesoid X receptor; TMA, trimethylamine; TLR4, Toll-like receptor 4.

### 5.1 Acute lung injury (ALI) and acute respiratory distress syndrome (ARDS)

Sepsis-induced acute lung injury (ALI) and acute respiratory distress syndrome (ARDS) are major complications marked by impaired gas exchange, diffuse pulmonary inflammation, increased vascular permeability, and non-cardiogenic pulmonary edema (Ortiz et al., 2019; Zhang et al., 2024). The gut-lung axis provides a mechanistic framework for understanding how intestinal dysbiosis and barrier failure may contribute to pulmonary injury during sepsis. When intestinal barrier integrity is compromised, gut-derived microbial products, inflammatory mediators, and damage-associated molecular patterns may disseminate systemically or enter the circulation through mesenteric lymphatics, thereby amplifying pulmonary inflammation (Dickson et al., 2016; Stanley et al., 2016; Zhou and Liao, 2021; Ziaka and Exadaktylos, 2024). These signals are recognized by pattern recognition receptors on innate immune cells, including macrophages, neutrophils, and dendritic cells, triggering pro-inflammatory responses that contribute to lung epithelial injury, endothelial dysfunction, vascular leakage, and multiple organ dysfunction syndrome (MODS) (Li and Wu, 2021; Zhou and Liao, 2021). This process may become self-reinforcing, as systemic inflammation and organ dysfunction further compromise intestinal barrier integrity and sustain gut-derived inflammatory signaling.

In addition to barrier-derived inflammatory signals, microbiota-derived metabolites may provide a second route linking intestinal dysbiosis to lung injury by regulating innate immune activation and neutrophil survival. Yang et al. (2025b) used CLP septic rats, CLP- or LPS-induced mouse ALI models, and MH-S alveolar macrophages to define a gut microbiota-Pro-Leu-NOD2/NF-κB pathway in sepsis-associated ALI. CLP enriched *Bacteroides* and *Escherichia-Shigella* and increased fecal Pro-Leu levels (*p* < 0.001); functionally, Pro-Leu aggravated lung injury at 10 mg/kg and dose-dependently enhanced inflammatory cytokine expression in MH-S cells at 10–50 μM by activating C/EBPβ/NOD2/NF-κB/NLRP3 signaling, while NOD2 inhibition with GSK717 attenuated these effects. Although this study supports a gut microbiota-Pro-Leu-NOD2/NF-κB–lung inflammation axis, its clinical relevance is limited by the absence of human sepsis validation and by reliance on correlative microbiome metabolite analyses and exogenous Pro-Leu supplementation. Xuan et al. (2024) integrated BALF neutrophil transcriptomics from patients with sepsis-induced ARDS, CLP mouse models, 16S rRNA sequencing, SCFA profiling, acetate intervention, FABP4 inhibition, ER-stress modulation, and neutrophil depletion to define an acetate–FFAR2–FABP4–neutrophil axis, in CLP mice, gut microbial composition differed from sham controls (ANOSIM *R* = 0.2, *p* = 0.01), serum acetate was increased and strongly correlated with neutrophil proportion (*r* = 0.9093, *p* = 0.0003), and acetate downregulated neutrophil FABP4 via FFAR2, delayed neutrophil apoptosis, and promoted inflammatory cytokine release, epithelial injury, and ARDS progression. The study links dysbiosis-induced acetate depletion to FFAR2/FABP4-mediated ER stress and neutrophil apoptosis, but its clinical relevance is weakened by mainly transcriptomic human evidence and limited direct validation of acetate and FABP4 causality in patients. These mechanisms suggest that the gut-lung axis contributes to ALI/ARDS mainly through inflammatory signal amplification and neutrophil-centered tissue injury rather than through a single microbial pathway.

### 5.2 Acute kidney injury (AKI)

Acute kidney injury (AKI) is one of the most frequent organ complications of sepsis and is closely associated with higher in-hospital mortality and longer hospital stays in critically ill patients (Hoste et al., 2015; Liu et al., 2020; Zarbock et al., 2023; Pais et al., 2024). Sepsis is frequently accompanied by gut microbiota dysbiosis and intestinal barrier disruption, both of which are increasingly recognized as contributors to sepsis-associated AKI (SA-AKI) (Kullberg et al., 2021; Winner et al., 2025). The gut-kidney axis is bidirectional: kidney injury can disrupt microbial ecology and epithelial integrity, whereas gut dysbiosis can increase harmful metabolites and inflammatory signals that worsen renal injury (Meijers and Evenepoel, 2011; Evenepoel et al., 2017).

Microbiota-derived signals do not act uniformly on the kidney; their effects depend on disease context, microbial composition, and the balance between protective and injurious metabolites. Experimental studies have shown that fecal microbiota transplantation from healthy mice attenuates renal injury, suggesting that the normal gut microbiota produces renoprotective factors (Nakade et al., 2018). Al-Harbi et al. (2018) and Tian et al. (2025) used LPS-induced SA-AKI mouse models to show that SCFAs exert renal protection through distinct mechanisms, acetate supplementation reduced serum creatinine (Scr), blood urea nitrogen (BUN), renal myeloperoxidase activity, lipid peroxidation, and tubular injury by suppressing T-cell HDAC–NOX2/ROS signaling, whereas butyrate supplementation, whose renal levels negatively correlated with Scr (*r* = −0.92, *p* = 0.00013) and BUN (*r* = −0.72, *p* = 0.018), alleviated renal inflammation and injury by inhibiting STING/NLRP3/GSDMD-mediated pyroptosis. These studies support a renoprotective role of SCFAs in SA-AKI, but their evidence is mainly based on LPS models and *in vitro* interventions, lacking human sepsis validation and direct proof that exogenous SCFA supplementation reflects microbiota-derived causal effects *in vivo*. In animal models of hypoxia-induced tubular injury, gut-derived D-serine has also been reported to reduce hypoxia-induced tubular injury and promote tubular cell proliferation during recovery (Nakade et al., 2018). However, these effects are context-dependent, as broad-spectrum antibiotic-mediated microbiota depletion has also been shown to attenuate renal ischemia–reperfusion injury by limiting the maturation of renal resident macrophages and bone marrow-derived monocytes (Emal et al., 2017).

In contrast to these potentially protective microbial metabolites, intestinal barrier dysfunction may provide an additional link between sepsis and renal injury by facilitating the systemic entry of gut-derived uremic toxins, which can further aggravate renal dysfunction (Dicu-Andreescu et al., 2024; Lee et al., 2024; Tsuji et al., 2024). These toxins include indoxyl sulfate (IS), p-cresyl sulfate (PCS), TMAO, indole-3-acetic acid, and phenylacetylglutamine. Among them, IS and PCS are protein-bound metabolites generated by anaerobic bacterial metabolism of amino acids in the gut and are poorly cleared by conventional hemodialysis because of their high protein-binding affinity (Rossi et al., 2015; Favretto et al., 2017; Liang et al., 2018; Chen et al., 2019). IS and PCS, have been linked to renal inflammation and tubular injury in AKI animal models, human cohort studies and translational gut-kidney axis research (Wu et al., 2011; Cheng et al., 2020; Tsuji et al., 2024). These mechanisms suggest that SA-AKI is linked to three related processes: loss of protective microbial signals, accumulation of gut-derived toxins, and barrier-driven inflammation.

### 5.3 Liver injury and liver dysfunction

Sepsis-associated liver injury (SALI) is a clinically important manifestation of organ dysfunction and is linked to poor outcomes in sepsis (Nesseler et al., 2012; Woźnica et al., 2018). The gut-liver axis provides a direct anatomical and functional route through which intestinal dysbiosis may amplify hepatic injury. Evidence from both clinical observational studies in critically ill patients and experimental sepsis models suggests that disruption of the intestinal epithelial barrier facilitates the translocation of gut-derived microorganisms and microbial products into the portal circulation and systemic compartment, thereby increasing hepatic exposure to pathogen-derived molecules such as lipopolysaccharide (LPS) (Sun et al., 2020; Kassa et al., 2021; Shahid et al., 2024; Mak and Shekhar, 2025). These microbial signals can trigger inflammatory responses in hepatic immune and parenchymal cells, promoting cytokine release, oxidative stress, hepatocellular injury, and progression of liver dysfunction (Sun et al., 2020; Shahid et al., 2024).

Bile acids (BAs) represent another major link between gut microbial dysbiosis and SALI. BAs are synthesized from cholesterol in the liver, secreted into the intestine, modified by gut microbes in the distal small intestine and colon, and largely returned to the liver through enterohepatic circulation (Kiriyama and Nochi, 2021; Shao et al., 2021; Larabi et al., 2023). Under physiological conditions, BAs help shape the gut microbiota, exert antimicrobial activity, and contribute to intestinal barrier maintenance (Shao et al., 2021; Larabi et al., 2023). In a prospective adult sepsis cohort, Long et al. (2023) analyzed 109 fecal samples from 23 septic patients, 16 nonseptic ICU patients, and 10 healthy controls, showing depletion of Firmicutes and SCFA-producing genera, expansion of Proteobacteria and opportunistic pathogens, reduced SCFAs and secondary bile acids, and increased primary bile acids; a model combining SOFA score, Klebsiella, taurocholic acid, and butyric acid predicted sepsis mortality with an AUC of 98.6% (*p* < 0.001). Kean et al. (2022) similarly showed bile acid dysmetabolism in 70 critically ill children, with increased primary bile acids at days 1–3 and 4–7 (both *p* < 0.001), reduced secondary bile acids, and a CA ratio shift from 0.9 in controls to 4.4 and 5.9 during hospitalization. Mechanistically, Li et al. (2022) showed that in CDAHFD-induced liver injury rats, reduced conjugated bile acids coincided with increased gut permeability and portal LPS exposure as early as 48 h, whereas inhibition of microbial bile acid deconjugation with AAA-10 (a gut-restricted microbial bile salt hydrolase inhibitor that blocks bile acid deconjugation) restored conjugated bile acids, reduced portal LPS, and attenuated barrier dysfunction and hepatic inflammation. Together, these studies support a candidate gut-liver axis in which sepsis- or critical illness-associated gut dysbiosis is accompanied by bile acid dysmetabolism, while mechanistic evidence from diet-induced liver injury models suggests that microbial bile acid deconjugation can weaken conjugated bile acid-mediated epithelial barrier protection, increase intestinal permeability and portal LPS exposure, and thereby amplify hepatic inflammation. However, direct causal validation of this complete dysbiosis-bile acid-barrier dysfunction-liver inflammation pathway in human sepsis-associated liver injury remains lacking. Evidence from rodent liver injury models suggests that dysregulated BA signaling may further disturb farnesoid X receptor (FXR)-related pathways, aggravating hepatic inflammation, oxidative stress, and hepatocellular injury (Ommati et al., 2021; Punzo et al., 2024). Thus, portal microbial translocation and BA dysregulation form two convergent routes through which gut dysfunction may worsen SALI. Collectively, the gut-liver axis may promote SALI through portal microbial product exposure, LPS-driven inflammation, disrupted BA/FXR signaling, oxidative stress, and hepatocellular injury.

### 5.4 Sepsis-associated encephalopathy (SAE)

Sepsis-associated encephalopathy (SAE) is defined as a diffuse cerebral dysfunction occurring in the setting of sepsis, characterized by altered mental status ranging from delirium to coma, in the absence of direct central nervous system infection, structural brain lesions, or other primary causes of encephalopathy (Li et al., 2025). Gut microbiota dysbiosis plays a critical role in the pathogenesis of SAE through disruption of intestinal barrier integrity. In septic mice, alterations in gut microbial composition lead to a reduction in tight junction proteins, including ZO-1, occludin, and claudins, resulting in increased intestinal permeability and the formation of a “leaky gut” (Yoseph et al., 2016). Gut-derived LPS have been demonstrated in experimental sepsis models to enter the systemic circulation or propagate systemically, where they activate innate immune pathways, particularly TLR4-mediated inflammatory signaling (Tan et al., 2022a). Circulating LPS activates the TLR4/NF-κB signaling pathway and promotes NLRP3 inflammasome activation, thereby amplifying systemic inflammatory cascades (Wei et al., 2025). Sustained systemic inflammation during sepsis can activate cerebral microvascular endothelial cells and impair endothelial barrier function, resulting in tight junction disruption, blood–brain barrier (BBB) leakage, and increased permeability (Erikson et al., 2020; Haileselassie et al., 2020). Supported by preclinical mechanistic studies and corroborated by human autopsy-based associative evidence, BBB disruption during sepsis may allow peripheral inflammatory mediators and neurotoxic molecules to gain access to the central nervous system, where they contribute to microglial activation and astrocytic reactivity, thereby amplifying neuroinflammation (Su et al., 2025). This activation results in excessive release of pro-inflammatory cytokines, oxidative stress, neuronal injury, and ultimately contributes to the development and progression of SAE (Guo et al., 2024a; Guo et al., 2024b).

Gut microbiota dysbiosis may also contribute to the development of SAE by disrupting the production of microbiota-derived metabolites, particularly SCFAs and tryptophan-derived indole metabolites (Fang et al., 2022; Liao et al., 2022). Liao et al. (2022) used a CLP-induced SAE mouse model with SCFA pretreatment, 16S rRNA sequencing, fecal SCFA profiling, and GPR43 antagonism by GLPG0974 to define a microbiota–SCFA–GPR43 neuroprotective pathway, in CLP mice, fecal acetate and propionate were markedly reduced compared with sham mice (acetate: 0.57 ± 0.09 vs. 2.00 ± 0.24 mg/g, *p* < 0.001; propionate: 0.32 ± 0.06 vs. 0.66 ± 0.12 mg/g, *p* = 0.002), accompanied by depletion of SCFA-producing bacteria, including *Allobaculum*, *Bacteroides*, and *Bifidobacterium*; SCFA pretreatment at 500 mg/kg twice daily restored acetate and propionate levels, improved Morris water maze performance, and reduced hippocampal IL-1β, IL-6, and TNF-α, whereas GLPG0974 reversed these cognitive and anti-neuroinflammatory effects. The findings support a protective role for SCFA-GPR43 signaling in SAE, but causal and translational confidence is limited by mouse pretreatment design, lack of GPR43-knockout validation, absent brain SCFA data, and no post-sepsis therapeutic assessment. SCFAs also preserve intestinal and blood brain barrier integrity by upregulating tight junction proteins such as ZO-1 and occludin in the intestine, brain tissue, and LPS-treated cerebro-microvascular cells, thereby limiting the entry of peripheral inflammatory and neurotoxic signals into the central nervous system (Li et al., 2023). In addition, SCFAs can attenuate behavioral impairment and neuronal degeneration in SAE mice by reducing brain IL-1β and IL-6 levels and suppressing JNK/NF-κB-related neuroinflammatory signaling (Liu et al., 2021a). Butyrate deficiency may further increase SAE susceptibility, whereas sodium butyrate reduces hippocampal oxidative stress, inhibits ROS generation and iNOS expression in LPS-stimulated primary microglia, and activates the GPR109A/Nrf2/HO-1 antioxidant pathway (Zhang et al., 2022). Fang et al. (2022) used CLP-induced SAE mice, 16S rRNA sequencing, fecal metabolomics, FMT, IPA supplementation, and LPS-stimulated primary microglia to define a gut microbiota–IPA–AhR–microglial NLRP3 pathway, showing that SAE-susceptible mice, defined by neurological score ≤6 at 36 h after CLP, had reduced microbial diversity and Enterobacteriaceae enrichment (PCA *R*^2^ = 0.2083, *p* = 0.013), that FMT from susceptible mice caused 100% mortality within 48 h and higher serum/cortical IL-1β and TNF-α than FMT from resistant mice, and that IPA, enriched in SAE-resistant mice, improved 7-day survival and neurobehavioral outcomes while suppressing microglial NLRP3 activation, caspase-1 cleavage, and IL-1β release through an AhR-dependent mechanism. Despite providing mechanistic support for a microbiota-derived IPA mechanism in SAE susceptibility, the study is limited by its reliance on CLP mice and *in vitro* microglial assays, the absence of human SAE validation, and the lack of AhR-knockout or microglia-specific evidence to confirm the precise target of IPA.

Besides, indirect preclinical evidence suggests that antibiotic-induced gut dysbiosis may facilitate intestinal overgrowth and gut-blood–brain translocation of *Klebsiella pneumoniae*, thereby triggering neuroinflammation and neurobehavioral impairment (Park et al., 2024a). Xi et al. (2021) used a CLP-induced SAE rat model combined with FMT, 16S rRNA sequencing, GW4869 (an exosome secretion inhibitor)- mediated inhibition of exosome release, hippocampal IL-1β/IL-1β antagonist intervention, and IEC-derived exosome stimulation of macrophages and H19-7 neurons, showing that IEC-derived exosomes promoted mesenteric lymph node macrophage M1 polarization and IL-1β release, thereby aggravating hippocampal neuronal injury in an autophagy-dependent manner. Although this study provides mechanistic evidence linking gut dysbiosis to SAE-related neuronal injury through IEC-derived exosomes and IL-1β, its reliance on CLP rats and cell models, lack of human validation, non-specificity of GW4869, and unidentified exosomal cargo limit its mechanistic specificity and clinical translatability. Collectively, current evidence suggests that gut dysbiosis acts as a multifaceted upstream amplifier of SAE by integrating intestinal barrier failure, microbial and metabolite-derived signals, pathobiont translocation, and gut immune-exosomal communication into systemic inflammation, BBB dysfunction, glial activation, neuroinflammation, and neuronal injury.

### 5.5 Myocardial dysfunction

Sepsis-induced myocardial dysfunction (SIMD) is a major complication of sepsis, with reported prevalence varying widely and approaching 50% in patients with septic shock, depending on diagnostic criteria and assessment methods (Lin et al., 2022; Lukić et al., 2024). Chen et al. (2022) and Deng et al. (2023) used small single-center case–control designs to compare septic patients with and without SIMD/sepsis-induced cardiomyopathy (SICM), integrating 16S rRNA sequencing or metagenomic/virome profiling: Chen et al. (2022) included 18 SIMD and 18 non-SIMD patients and found higher SOFA scores, high-sensitivity cardiac troponin I (hs-cTnI) levels, and lower left ventricular ejection fraction (LVEF) in SIMD, together with enrichment of Proteobacteria, Enterobacteriaceae, and *Klebsiella variicola*, depletion of Bacteroidetes, Bacteroidaceae, and *Bacteroides vulgatus*, and moderate diagnostic performance of *K. variicola* for SIMD (AUC 0.762, 95% CI 0.569–0.936); Deng et al. (2023) similarly included 15 SICM and 16 non-SICM patients and reported higher SOFA scores (10 [7–14] vs. 6 [5–10], *p* = 0.005), hs-cTnI (0.334 vs. 0.029 ng/mL, *p* < 0.001), lactate levels (4.40 vs. 2.50 mmol/L, p < 0.001), lower LVEF (38.6% vs. 64.3%, p < 0.001), and diagnostic value of *Cronobacter* and *Cronobacter* phage for SICM (AUC 0.712 and 0.775, respectively). In contrast, the gut microbiota of patients without SIMD shows greater ethylbenzene degradation capacity, which may be associated with reduced myocardial injury. Together, these studies suggest that gut dysbiosis may contribute to sepsis-related myocardial injury and risk stratification, but their small, single-center, observational designs, limited sampling time points, and lack of functional validation make it difficult to determine whether these microbial changes are causal, consequential, or merely reflect disease severity and antibiotic exposure. Although the causal link remains incompletely defined, microbiota-derived metabolites and gut-derived inflammatory signals appear to contribute to SIMD pathogenesis.

This association may be mediated through convergent metabolic, inflammatory, and mitochondrial pathways. In critical illness and related inflammatory settings, gut microbes generate trimethylamine, which is converted in the liver to trimethylamine N-oxide (TMAO), a metabolite linked to adverse cardiovascular outcomes (Zhen et al., 2023; Khan et al., 2024). However, direct sepsis-specific evidence for this pathway remains limited. Human observational and experimental mechanistic evidence suggests that gut dysbiosis disrupts intestinal barrier integrity and promotes translocation of lipopolysaccharide (LPS) into the circulation (Violi et al., 2023; Di Vincenzo et al., 2024). Established innate immune and experimental evidence indicates that circulating LPS can activate TLR4-dependent inflammatory signaling in immune cells and cardiomyocytes, in sepsis-induced myocardial injury animal models, STING- and NLRP3-related pathway activation has been implicated in cytokine release, oxidative stress, apoptosis, and myocardial damage, although direct human evidence linking gut dysbiosis-derived LPS to this pathway remains limited (Nong et al., 2023; Basak and Akashi-Takamura, 2024; Long et al., 2024; Xu et al., 2024). Clinical observational and translational evidence suggests that sepsis can alter bile acid metabolism, animal and *in vitro* cardiovascular studies further indicate that excessive bile acid-FXR signaling may contribute to conduction abnormalities, hypertrophic remodeling, cardiomyocyte apoptosis, and impaired contractility (Zhang et al., 2023b; Zhou et al., 2025). These studies indirectly support a possible link between sepsis-related gut dysbiosis, bile acid dysmetabolism, and septic cardiomyopathy; however, because the evidence is mainly review-based or derived from an LPS organic injury model, direct causal validation of the gut microbiota-bile acid-cardiac dysfunction pathway in sepsis remains lacking. Animal and in vitro studies of sepsis-induced myocardial injury implicate oxidative stress, altered mitophagy, and mitochondrial dysfunction as key mechanisms of cardiomyocyte damage (Du et al., 2021). Microbiota dysregulation may interact with these pathways through gut-derived inflammatory or metabolic signals, although direct evidence linking dysbiosis to mitochondrial injury in septic cardiomyopathy remains limited (Hu et al., 2024). Compared with the gut-lung, gut-kidney, and gut-liver axes, evidence for the gut-heart axis remains less mature. Current data support a plausible link, but stronger causal and clinical studies are still needed.

## 6 Microbiota-targeted therapeutic strategies for sepsis and sepsis-associated organ injury

### 6.1 Therapeutic rationale for targeting the gut microbiota

Sepsis management remains centered on timely infection control, antimicrobial therapy, hemodynamic resuscitation, and organ support (Evans et al., 2021). Microbiota-targeted therapy should therefore be viewed as a mechanism-informed adjunctive strategy rather than a replacement for standard sepsis care. Its therapeutic rationale comes from the observation that gut dysbiosis in critical illness is not merely a secondary phenomenon, but may actively contribute to sepsis progression by weakening colonization resistance, disrupting epithelial barrier integrity, promoting microbial product translocation, altering microbial metabolites, and amplifying systemic immune dysfunction (Cho et al., 2024; Shimizu et al., 2025).

These mechanisms create several potentially treatable pathways. Restoration of microbial diversity may help re-establish colonization resistance and limit pathobiont expansion; reinforcement of the intestinal barrier may reduce systemic exposure to gut-derived microbial products; and modulation of microbial metabolites, especially short-chain fatty acids and other immunometabolic signals, may attenuate inflammatory injury and regulated cell death. Causal-supporting animal evidence from antibiotic-treated CLP septic mice shows that fecal microbiota transplantation and SCFA supplementation reduce sepsis mortality by remodeling antibiotic-induced gut microbiota disturbances, improving epithelial barrier integrity, and attenuating inflammatory and pyroptosis-related responses (Lou et al., 2022). In critically ill patients, disruption of the gut microbiota-immune metasystem has also been associated with nosocomial infections, supporting the idea that microbiome and immune phenotypes may influence host defense and clinical outcomes (Schlechte et al., 2023).

Accordingly, microbiota-targeted strategies in sepsis can be grouped into several therapeutic categories: microbial community restoration, barrier repair, metabolite- or postbiotic-based modulation, microbiota-modulating pharmacological or natural compounds, and organ-axis-oriented interventions. However, these approaches differ markedly in evidence level and clinical readiness. Most mechanistic data remain preclinical, while clinical studies are heterogeneous in patient populations, microbial formulations, timing, and endpoints. This section reviews how gut microbiota-related mechanisms may guide adjunctive therapeutic strategies for sepsis and sepsis-associated organ injury, while noting that these approaches are not yet established treatments. To avoid treating heterogeneous interventions as equivalent, we classify microbiota-related therapeutic strategies according to their biological nature and evidence level: live biotherapeutics and microbial community restoration, microbial metabolites or postbiotics, host-directed drugs with microbiome-associated effects, and exploratory natural-product or nanovesicle-based interventions. This classification is summarized in Table 2 and is used below to distinguish mechanistic proof-of-concept from clinically more mature approaches.

**Table 2:** Evidence hierarchy of microbiota-related therapeutic strategies in sepsis and sepsis-associated organ injury.

Therapeutic category	Representative interventions	Main evidence base	Relationship to gut microbiota	Reference
Live biotherapeutics and microbial community restoration	FMT, probiotics, synbiotics	FMT: preclinical sepsis models probiotics and synbiotics: preclinical sepsis models and heterogeneous critically ill populations	Direct restoration or supplementation of microbial communities	(Chen et al., 2020; Assimakopoulos et al., 2021; Han et al., 2022; Lou et al., 2022; Sharif et al., 2022; Wang et al., 2022; Lee et al., 2023; Lou et al., 2024)
Microbial metabolites and postbiotics	SCFAs, D-serine, 4-HPA, 3-HB, β-HB, betulinic acid	Mainly animal and cell-based studies in sepsis-associated organ injury or related kidney injury/liver injury models	Microbiota-derived or microbiota-regulated functional mediators	(Nakade et al., 2018; Lou et al., 2022; Kim et al., 2023; An et al., 2024; Hu et al., 2025; Tang et al., 2025b)
Host-directed drugs with microbiome-associated effects	Metformin	Preclinical sepsis-related liver injury and neuroinflammation models with microbiome and metabolomic profiling	Conventional drug with both direct host effects and microbiota-associated changes	(Liang et al., 2022; Zhao et al., 2022)
Natural compounds and herbal formulations	Nobiletin, Sinomenine, Naringin dihydrochalcone, YZC extract, Sini decoction, Qi Huang Fang	Preclinical animal studies with 16S sequencing, barrier assessment, metabolomics, or inflammatory pathway analysis	Microbiota-modulating rather than microbiota-derived interventions	(Song et al., 2021; Huang et al., 2023; Cao et al., 2024; He et al., 2024; Tang et al., 2025b)
Plant-derived nanovesicles	Epimedium-derived exosome-like nanovesicles	Preclinical evidence in sepsis-associated acute kidney injury	Remodel gut microbiota and gut-kidney signaling	(Yang et al., 2026)

This table summarizes representative microbiota-related therapeutic strategies according to their biological category, main evidence base and relationship to the gut microbiota. Evidence levels were interpreted according to the source and maturity of available data, including clinical studies, preclinical animal models, microbiota-transfer experiments, metabolomic analyses, and in vitro mechanistic studies. Interventions listed as microbiota-derived or microbiota-regulated metabolites should be distinguished from host-directed pharmacological agents, natural compounds, herbal formulations, and plant-derived nanovesicles, which may modulate the microbiota but are not necessarily produced by gut microbes. Most strategies remain experimental or hypothesis-generating and should not be interpreted as established adjunctive therapies for sepsis. FMT, fecal microbiota transplantation; 3-HB, 3-hydroxybutyrate; 4-HPA, 4-hydroxyphenylacetic acid; β-HB, β-hydroxybutyrat; YZC, Yazhicao.

### 6.2 Microbiota restoration and barrier repair

Restoring disrupted microbial communities is a direct strategy for limiting gut-derived inflammatory amplification in sepsis. Fecal microbiota transplantation (FMT) aims to reconstruct microbial diversity, restore colonization resistance, reduce pathobiont expansion, and reinforce epithelial barrier integrity. In experimental sepsis models, FMT has been reported to reshape gut microbial composition, improve survival, reduce systemic inflammatory responses, and preserve mucosal barrier function. Assimakopoulos et al., (2021) used a CLP-induced rat sepsis model with delayed FMT at 6 h, showing that FMT improved 7-day survival from 0 to 50% (*p* < 0.001), restored ileal mucosal thickness and crypt cleaved caspase-3-positive cells, increased occludin and claudin-1 expression and reduced systemic endotoxemia; Lou et al. (2022) further combined fecal 16S rRNA sequencing in 22 septic patients and 12 controls with antibiotic-disrupted CLP mice, showing lower serum SCFAs in sepsis patients (*p* < 0.0001), Proteobacteria enrichment and Firmicutes depletion, and that FMT or SCFA supplementation improved survival, bacterial clearance, occludin expression, organ injury, and NLRP3/GSDMD-N/IL-18/IL-1β-mediated pyroptosis. Another murine study further suggested that FMT protects the intestinal mucosal barrier by reconstructing the gut microbiota during sepsis (Gai et al., 2021). FMT and SCFAs may confer protection in sepsis by restoring microbial balance, strengthening barrier function, and limiting systemic inflammation, but their clinical translation remains constrained by short-term animal evidence, undefined active components, unclear causal taxa or metabolites, and limited human validation.

Probiotics, prebiotics, and synbiotics provide more defined approaches to microbial community restoration, although their effects are highly strain-, substrate-, and host-dependent. Experimental studies suggest that selected probiotics can improve sepsis outcomes by reshaping gut microbial composition and metabolic profiles. Chen et al. (2020) administered *Lactobacillus rhamnosus* GG before CLP for 4 weeks and showed improved 7-day survival (*p* = 0.029), reduced bacteremia, partial restoration of microbial diversity, increased acetate, and correction of sepsis-associated lipid and bile acid disturbances; Han et al. (2022) treated LPS-induced septic mice with *Lactobacillus johnsonii* 6,084, increasing 7-day survival from approximately 20 to 50% and reducing serum IL-1β/TNF-*α*, tissue IL-1β/TNF-α/IL-6 and histological lung, liver, kidney, and intestinal injury while partly restoring sepsis-altered Bacteroidetes, Proteobacteria, and genus-level dysbiosis. These studies suggest that selected Lactobacillus strains may improve sepsis outcomes by modulating gut microbiota, metabolic imbalance, and systemic inflammation; however, their small animal-based designs, strain-specific effects, lack of human validation, and limited causal evidence for key taxa or metabolites restrict clinical translation.

Clinical evidence for probiotics and synbiotics is more heterogeneous. Recent systematic reviews and meta-analyses in critically ill patients suggest that probiotics or synbiotics may reduce selected infection-related outcomes, including ventilator-associated pneumonia, diarrhea, or septic complications; however, effects on mortality and broader sepsis outcomes remain inconsistent (Sharif et al., 2022; Wang et al., 2022; Lee et al., 2023; Lou et al., 2024). Therefore, evidence from general ICU populations, postoperative patients, neonates, or preterm infants should not be directly extrapolated to adult sepsis or septic shock. The therapeutic value of probiotics and synbiotics is likely to depend on baseline microbial ecology, antibiotic exposure, disease severity, immune status, timing of administration, and the specific strains or substrates used.

Importantly, therapeutic modulation of the gut microbiota in sepsis must be framed by safety considerations from the outset. Septic patients frequently have impaired epithelial barrier integrity, immune dysfunction, indwelling catheters, broad-spectrum antibiotic exposure, and a high baseline risk of bloodstream infection (Rhee et al., 2024; Varkila et al., 2024). Therefore, live microbial interventions, including FMT, probiotics, and synbiotics, should not be viewed as broadly benign microbiota-restorative approaches. Their potential benefits must be balanced against risks of pathogen transmission, microbial translocation, bacteremia, or fungemia, and should be evaluated with strict donor or strain screening, patient selection, and adverse-event monitoring.

Barrier repair is the key mechanistic link connecting microbiota restoration to sepsis control. A restored microbial ecosystem may reduce luminal pathogen overgrowth, increase colonization resistance, preserve mucus and tight junction integrity, and limit translocation of bacteria, LPS, and other microbial products into systemic compartments. However, future studies should move beyond broad clinical endpoints and include direct measures of barrier function, microbial product translocation, strain-level engraftment, and microbiome-derived metabolic recovery. At present, FMT, probiotics, prebiotics, and synbiotics are best considered experimental adjuncts rather than established sepsis therapies.

### 6.3 Microbial metabolites and postbiotic-based strategies

Microbial metabolites provide a functional link between gut dysbiosis and host inflammatory injury. Compared with live microbial therapies, metabolite- or postbiotic-based strategies may offer a more controllable approach because they do not require administration of replicating organisms. Among these metabolites, SCFAs, particularly butyrate, have received the most attention. Experimental studies show that microbiota-derived butyrate contributes to intestinal epithelial homeostasis, regulates barrier-related cytoskeletal and tight junction pathways, and attenuates cytokine-induced epithelial barrier dysfunction (Tabat et al., 2020; Wang et al., 2020a; Huang et al., 2021). Butyrate also suppresses inflammatory immune activation and modulates cytokine release in epithelial-immune cell co-culture systems (Korsten et al., 2023). These findings support SCFAs as functional mediators of gut microbiota-host communication rather than passive markers of microbial fermentation.

In sepsis, SCFA-related strategies may influence both systemic inflammation and organ injury. In a mouse model of sepsis, fecal microbiota transplantation and SCFAs reduced mortality by remodeling antibiotic-induced gut microbiota disturbances, suggesting that restoration of microbial metabolic output may contribute to improved host resilience (Lou et al., 2022). Animal and cell-based evidence in SA-AKI suggests that SCFAs may regulate renal inflammation through immune and cell-death pathways. Acetate ameliorated renal injury by suppressing NOX2/ROS signaling in T cells in a CLP-induced sepsis-associated AKI mouse model, whereas butyrate inhibited STING-GSDMD related pyroptotic signaling in an LPS-induced SA-AKI mouse model and LPS-treated HK-2 renal tubular epithelial cells (Al-Harbi et al., 2018; Tian et al., 2025).

Other microbiota-derived or microbiota-regulated metabolites have also shown organ-protective potential. An et al. (2024) combined CLP-induced SA-AKI mice, gut microbiota depletion, 4-HPA tracing, renal metabolomics, LPS-stimulated HK-2 cells, ARC (apoptosis repressor with caspase recruitment domain) knockdown, and plasma analysis from 29 septic patients, showing that renal metabolomics detected 652 metabolites with 79 altered after CLP and that gut-derived 4-HPA was increased and correlated with renal injury in mice and patients, including renal 4-HPA with Cr/BUN (*r* = 0.5250 and 0.5582; *p* = 0.0174 and 0.0105) and plasma 4-HPA with Cr, BUN, eGFR, and SOFA score (*r* = 0.5369, 0.4597, −0.5236, and 0.5240; *p* = 0.0027, 0.0121, 0.0036, and 0.0086), while mechanistically 4-HPA upregulated ARC by 1.694-fold, enhanced ARC-RIPK1 interaction, and inhibited RIPK1/RIPK3/MLKL-mediated necroptosis to alleviate SA-AKI. Overall, this study supports a renoprotective role of gut-derived 4-HPA in SAKI, its clinical relevance is limited by small-scale correlative human data, unidentified 4-HPA-producing microbes, and the predominance of pretreatment-based animal experiments. Additionally, evidence from non-sepsis ischemia/reperfusion-induced AKI mouse models and hypoxia-injured tubular epithelial cells suggests that gut microbiota-derived D-serine may support tubular protection and repair (Nakade et al., 2018). β-Hydroxybutyrate has also been reported to reduce tubular apoptosis and inflammatory responses in LPS-induced mouse model of SA-AKI, further supporting ketone-body-related metabolic modulation as a potential organ-protective strategy (Kim et al., 2023). In CLP- and LPS-induced mouse models of SALI, time-restricted feeding reshapes the gut microbiota and increases 3-hydroxybutyrate, which inhibits hepatocyte ferroptosis through the PI3K/AKT/mTOR/LPIN1 pathway (Hu et al., 2025). In CLP-induced mouse model of SALI, gut microbe-derived betulinic acid alleviates sepsis-induced acute liver injury by inhibiting macrophage NLRP3 inflammasome activation, linking microbial metabolic remodeling to hepatic immune regulation (Tang et al., 2025b).

Microbial metabolites may affect several injury pathways, including barrier dysfunction, immune activation, oxidative stress, and regulated cell death. However, metabolite-based interventions remain at an early stage. Key unresolved issues include dose, route of administration, tissue specificity, timing relative to sepsis stage, and context-dependent effects in different host-microbiota states. Future studies should clarify whether these metabolites are causal mediators of protection, downstream markers of broader microbiota recovery, or both. For now, metabolite- and postbiotic-based strategies remain early-stage approaches that need stronger clinical testing.

### 6.4 Microbiota-modulating pharmacological and natural compounds

Beyond live microbial therapies and microbial metabolites, several pharmacological agents, natural compounds, herbal formulations, and plant-derived nanovesicles have been reported to modulate sepsis-associated organ injury through gut microbiota-related mechanisms. These interventions should be distinguished from microbial metabolites or postbiotics, because they are not directly produced by gut microbes. Instead, they may reshape microbial composition, restore microbial metabolic output, reinforce epithelial barrier integrity, reduce endotoxin translocation, or regulate downstream inflammatory and cell-death pathways.

Metformin is a representative pharmacological agent with microbiota-modulating properties. Liang et al. (2022) and Zhao et al. (2022) used CLP-induced septic rat models with metformin treatment, FMT, 16S rRNA sequencing, inflammatory profiling, and metabolomic analysis to examine a metformin-associated gut–organ protective mechanism: in aged SLI rats, Liang et al. (2022) showed that metformin reduced liver injury, hepatocyte apoptosis, ALT/AST elevation, hepatic inflammatory gene expression, and colonic barrier dysfunction, while shifting the microbiota from *Klebsiella*/*Escherichia-Shigella* enrichment toward *Bifidobacterium*, Muribaculaceae, *Parabacteroides distasonis*, and *Alloprevotella*; FMT from metformin-treated donors reproduced lower liver injury and barrier dysfunction in recipients, and correlation analysis linked *Klebsiella* positively with hepatic TNF-α and C-C motif chemokine ligand 4 (*r* = 0.98 and 0.89) but Muribaculaceae negatively with hepatic IL-6 and TNF-α (*r* = −0.97 and −0.84); Zhao et al. (2022) similarly showed that metformin reduced hippocampal injury, IBA1/GFAP activation, and TNF-α/IL-6/CXCL1 expression, increased SCFA-related taxa such as *Prevotella*, Muribaculaceae, and *Alloprevotella*, altered 29 predicted metagenomic functions, and reversed 30 fecal metabolites; Lactobacillaceae positively correlated with brain inflammatory markers (*r* = 0.96–0.97), whereas Muribaculaceae showed negative correlations (*r* = −0.96 to −0.98). Metformin may protect against sepsis-related organ injury through microbiota and metabolite remodeling, but animal-based evidence, absent human validation, unclear causal taxa or metabolites, and possible direct immunomodulation limit translational confidence.

Natural compounds may also influence sepsis-associated organ injury by reshaping gut microbial homeostasis and regulating downstream inflammatory pathways. Nobiletin, a polymethoxyflavone, alleviates sepsis-associated acute liver injury in CLP induced sepsis mouse model by modulating gut microbiota and activating the Nrf2-GPX4 pathway, thereby suppressing ferroptosis-related hepatic injury (Huang et al., 2023). In CLP induced sepsis mouse model, sinomenine has been shown to ameliorate septic acute lung injury by modulating gut homeostasis through the aryl hydrocarbon receptor/Nrf2 pathway (Song et al., 2021). Naringin dihydrochalcone alleviates sepsis-induced acute lung injury in CLP mouse model by improving gut microbial homeostasis and activating GPR18 receptor signaling (He et al., 2024). In addition, in LPS-induced septic acute lung injury mouse model, YZC (Yazhicao) extract attenuates septic acute lung injury by regulating intestinal microbiota and reducing TMAO/NLRP3 signaling (Cao et al., 2024). These natural-compound studies generally use CLP- or LPS-induced animal models and assess organ injury, microbial composition, barrier function, oxidative stress, and inflammatory signaling. Although they support microbiota-associated organ protection, most compounds are pleiotropic and may act directly on host pathways, antibiotic-depletion, FMT rescue, or metabolite-reconstitution experiments are needed to establish microbiota dependence.

Herbal formulations have also been explored in experimental sepsis models. Sini decoction was reported to ameliorate lung injury in septic mice by modulating gut microbiota composition (Wang et al., 2020b). Qi Huang Fang was reported to improve intestinal barrier function and modulate the gut microbiota in septic mice through NLRP3 inflammasome-mediated cellular pyroptosis (Shu et al., 2024). These findings are mechanistically interesting, but they should be interpreted cautiously because herbal formulations are chemically complex, and their microbiota-dependent effects are difficult to separate from direct pharmacological actions. Therefore, such interventions are better described as microbiota-modulating herbal formulations rather than microbiota-derived therapies.

Plant-derived nanovesicles represent an emerging but still preliminary strategy. Yang et al. (2026) isolated and characterized epimedium-derived exosome-like nanovesicles (ENVs) and, using LPS-injured HK-2 cells, a CLP-induced SA-AKI mouse model, *in vivo* imaging, 16S rRNA sequencing and multi-omics, showed that ENVs accumulated in injured kidneys, reduced renal injury scores (*p* < 0.01), apoptosis, ROS production, and TNF-α/IL-6/IL-1β expression (mainly *p* < 0.05–0.001), while partially restoring gut microbial diversity (*p* < 0.05), reversing SA-AKI-induced microbial structural shifts (PERMANOVA *p* < 0.01), reducing Enterobacteriaceae and Desulfovibrio enrichment (*p* < 0.01), increasing Lactobacillus and Bacteroides abundance (*p* < 0.05), promoting SCFA-related barrier protection, reducing LPS translocation, and suppressing TLR4/NF-κB signaling. Although this study provides multilayered mechanistic evidence for ENV-based S-AKI therapy, its reliance on small animal and *in vitro* models, undefined key bioactive components, and unvalidated causal taxa/metabolites limit clinical translation. At present, nanovesicle-based strategies should be regarded as experimental platforms rather than clinically mature microbiota-targeted therapies.

These agents add another possible route for microbiota-based therapy beyond FMT, probiotics, and microbial metabolites. Still, most evidence remains preclinical, and many compounds have pleiotropic effects that are not necessarily mediated by the gut microbiota. Future studies should determine whether their protective effects depend on microbial remodeling, microbial metabolites, epithelial barrier repair, or direct host-targeted actions. Barrier- and endotoxemia-oriented strategies may be more relevant to ALI/ARDS and SA-AKI, whereas metabolic and regulated cell-death pathways appear more relevant to SALI; evidence for gut-heart-directed therapy remains early.

### 6.5 Evidence grading, patient selection, and safety concerns

Despite increasing interest in microbiota-targeted therapy, the clinical readiness of different strategies varies substantially. Most interventions discussed above, including FMT, SCFAs, 4-hydroxyphenylacetic acid, 3-hydroxybutyrate, betulinic acid, metformin, nobiletin, and plant-derived nanovesicles, remain supported mainly by preclinical evidence. These findings should therefore be interpreted as mechanistic proof-of-concept rather than evidence for routine clinical use in sepsis. In contrast, probiotics and synbiotics have been tested more extensively in critically ill patients. Recent systematic reviews and meta-analyses suggest that probiotics or synbiotics may reduce selected infection-related outcomes, such as ventilator-associated pneumonia, diarrhea, ICU- or hospital-acquired pneumonia, or septic complications, but effects on mortality and broader sepsis outcomes remain inconsistent and depend on population, formulation, strain, dose, and trial design (Sharif et al., 2022; Wang et al., 2022; Lee et al., 2023; Lou et al., 2024). Evidence derived from neonates, perioperative patients, pancreatitis, or general ICU populations should not be directly extrapolated to adult sepsis or septic shock.

Patient selection is likely to determine whether microbiota-targeted interventions are beneficial, neutral, or harmful. Sepsis-associated dysbiosis is heterogeneous and evolves rapidly under the influence of antibiotics, vasopressors, enteral nutrition, immune status, organ support, and disease severity. Longitudinal studies in critical illness indicate that disruption of the gut microbiota-immune metasystem is associated with nosocomial infection risk, supporting the need for microbiome- and immune-informed stratification (Schlechte et al., 2023). Future trials should therefore consider microbial and host features, including loss of obligate anaerobes, depletion of SCFA-producing capacity, expansion of Enterobacteriaceae or other pathobionts, impaired intestinal barrier function, endotoxemia, antibiotic exposure, and immune phenotype. Strategies aimed at restoring colonization resistance may be more relevant after broad-spectrum antibiotic exposure or during recovery from dysbiosis, whereas metabolite- or postbiotic-based approaches may be safer in patients with severe immune dysfunction or marked epithelial barrier disruption.

Antibiotic exposure deserves particular consideration. Timely antimicrobial therapy remains central to sepsis care, and early antibiotic administration is associated with improved outcomes in appropriately selected patients (Leung et al., 2024). However, programs designed to shorten time-to-antibiotics may also increase unnecessary antibiotic exposure and related adverse effects, including further microbiome disruption (Donnelly et al., 2024). This creates a practical tension: antibiotics are essential for infection control, but they may also worsen dysbiosis, reduce colonization resistance, and impair engraftment of microbiota-based interventions. Future studies should therefore record antibiotic class, timing, duration, and de-escalation strategy when evaluating microbiota-targeted therapies.

Safety is a major barrier to clinical translation, especially for live microbial interventions. Septic patients often have impaired epithelial barriers, immune dysfunction, indwelling catheters, broad-spectrum antibiotic exposure, and high risk of bloodstream infection. FMT is generally considered feasible in selected settings, but pathogenic organism transmission remains a recognized concern, particularly in vulnerable or immunocompromised hosts (Park and Seo, 2021). Probiotic safety is also strain- and host-dependent. Cases of *Lactobacillus rhamnosus* sepsis, *Bacillus clausii* bacteremia, and *Saccharomyces* fungemia after probiotic administration have been reported, underscoring that live microbial products may translocate or cause bloodstream infection under specific clinical conditions (Chiang et al., 2021; García et al., 2021; Rannikko et al., 2021; Corredor-Rengifo et al., 2024). For this reason, future trials should include careful patient selection, strain- or donor-level quality control, pathogen screening, and adverse-event monitoring. Clinical endpoints should include infection, organ dysfunction, ICU stay, antibiotic exposure, and mortality. Until such data are available, microbiota-targeted strategies should remain experimental adjuncts.

## 7 Discussion and future directions

Current evidence indicates that gut dysbiosis participates in sepsis-related immune dysregulation, barrier failure, microbial translocation, and organ injury. Still, several uncertainties remain. It is difficult to distinguish causal dysbiosis from dysbiosis that reflects antibiotic exposure, shock severity, nutrition, or organ failure. Most organ-axis mechanisms are supported by animal models or small clinical cohorts, and their relevance to heterogeneous adult sepsis remains uncertain. Microbiota modulation may become an adjunct to standard sepsis care, but current evidence does not support routine clinical use.

From a translational perspective, the most clinically credible microbiota-related pathways are those that converge on intestinal barrier dysfunction, microbial product translocation, loss of colonization resistance, depletion of obligate anaerobes and SCFA-producing capacity, and pathobiont expansion. These pathways are supported by convergent evidence from critically ill cohorts, experimental sepsis models, and mechanistic studies, and they are biologically aligned with major clinical problems in sepsis, including secondary infection, endotoxemia, systemic inflammation, and organ dysfunction. In contrast, organ-specific metabolic pathways such as selected bile acid, TMAO, indole, or regulated cell-death mechanisms remain promising but less mature, especially when supported mainly by metabolomics associations or preclinical intervention studies.

The biomarkers closest to clinical translation are therefore likely to be composite rather than single microbial taxa. Candidate markers include loss of microbial diversity, depletion of obligate anaerobes or SCFA-producing taxa, Enterobacteriaceae or other pathobiont domination, reduced fecal or circulating SCFAs, altered bile acid or tryptophan-derived metabolite profiles, gut-derived toxins such as indoxyl sulfate, p-cresyl sulfate, and TMAO, and markers of barrier dysfunction or endotoxin exposure. Integrating these microbial and metabolic features with host immune phenotypes may be more informative than relying on single organisms or metabolites.

Therapeutic prioritization should also reflect evidence maturity and biological plausibility. Strategies aimed at restoring colonization resistance or microbial metabolic output may be most rational in patients with antibiotic-associated loss of obligate anaerobes, low SCFA-producing capacity, or pathobiont expansion. In contrast, chemically complex herbal formulations, plant-derived nanovesicles, and natural compounds with unclear microbiota dependence should remain lower-priority exploratory platforms until their active components, microbiota dependence, safety, and reproducibility are better defined. Live microbial interventions should be tested only in carefully selected patients because epithelial barrier failure, immune dysfunction, central venous catheters, and bloodstream infection risk may convert microbiota restoration from a potentially beneficial strategy into a safety concern.

Future studies should define which patients are most likely to benefit, when treatment should start, and which strategy should be used. Emerging multi-omics approaches may help move sepsis microbiome research from descriptive microbial signatures toward biomarker discovery and personalized intervention. Integrating shotgun metagenomics, metatranscriptomics, metabolomics, host transcriptomics, immune profiling, and clinical phenotyping could identify microbiota-based signatures linked to barrier dysfunction, endotoxemia, SCFA depletion, pathobiont expansion, immune dysregulation, and organ injury. Such integrated profiles may help distinguish patients who are more likely to benefit from colonization resistance restoration, metabolite- or postbiotic-based strategies, or safety-guided avoidance of live microbial therapies. However, these approaches require longitudinal sampling, standardized analytical pipelines, external validation, and clinically meaningful endpoints before they can guide precision microbiota-targeted therapy in sepsis. Key issues include treatment timing, dose, duration, delivery route, donor selection for FMT, strain specificity of probiotics, and the safety of live microbial products in patients with immune dysfunction or impaired barrier integrity (Tan et al., 2026). Longitudinal multi-omics studies, mechanistic experiments, and multicenter randomized trials are needed to identify causal microbiota-host interactions and predictive microbial or metabolic biomarkers. Future studies should also standardize sampling time points, DNA extraction procedures, sequencing strategies, 16S rRNA target regions or shotgun metagenomic depth, reference databases, and bioinformatic pipelines to improve reproducibility across sepsis microbiome studies. These steps will determine whether microbiota modulation can become a safe, phenotype-guided adjunctive strategy for sepsis care.
